# Epidemiology of diarrheagenic pathogens in acute hospitals in Singapore: a retrospective multicenter study

**DOI:** 10.1128/spectrum.02725-25

**Published:** 2025-11-12

**Authors:** Bryan M. H. Keng, Pandora Han, Adrian Low, Kyaw Thu Aung, Wei Ching Khor, Matthias Maiwald, Liat Hui Loo, Lynette Oon, Tse Hsien Koh, Ka Lip Chew, Crystal Wong, Wei Jie Seow, Yann Felix Boucher

**Affiliations:** 1 Saw Swee Hock School of Public Health, National University of Singapore and National University Health System 37580, Singapore, Singapore; 2 National Centre for Food Science, Singapore Food Agency 664266 , Singapore, Singapore; 3 Yong Loo Lin School of Medicine, National University of Singapore 37580, Singapore, Singapore; 4 School of Biological Sciences, Nanyang Technological University 54761, Singapore, Singapore; 5 Department of Food Science and Technology, National University of Singapore 37580, Singapore, Singapore; 6 KK Women’s and Children’s Hospital, Singapore, Singapore; 7 Duke-National University of Singapore Graduate Medical School, Singapore, Singapore; 8 Singapore General Hospital 37581, Singapore, Singapore; 9 National University Hospital 59053, Singapore, Singapore; 10 Changi General Hospital 26674, Singapore, Singapore; 11 Singapore Centre for Environmental Life Sciences Engineering, National University of Singapore 37580, Singapore, Singapore; 12 Infectious Diseases Translational Research Program, Department of Microbiology and Immunology, Yong Loo Lin School of Medicine, National University of Singapore 37580, Singapore, Singapore; Victorian Infectious Diseases Reference Laboratory, Melbourne, Australia

**Keywords:** multiplex PCR, acute infective diarrhoea, epidemiology

## Abstract

Acute infectious diarrhea imposes a substantial socioeconomic burden globally. In Singapore, information on etiological agents of diarrhea remains limited. Through analysis of data from public hospitals, this study presents key insights into the epidemiology of diarrheagenic pathogens. Two datasets comprising gastrointestinal multiplex polymerase chain reaction (PCR) panel results from adult and pediatric populations, drawn from five hospitals across several years, were collated. Stool samples included in the study were screened for 22 pathogens using a commercial PCR assay. Overall sample positivity, pathogen relative abundances, and co-detection rate were determined. Potential associations between co-detected pathogens were assessed via pairwise correlations. Additionally, exploratory analysis was undertaken to investigate seasonal patterns and climatic influence on pathogen detection. Sample positivity was 42.6% (3,212/7,543) in the adult data set and 52.1% (1,572/3,015) in the pediatric data set. Despite differences in ranking, the most frequently detected organisms in both groups were pathogenic *Escherichia coli*, *Clostridioides difficile*, *Salmonella*, and Norovirus. Differences in pathogen proportions between data sets reveal distinct epidemiological profiles in adults and children. Geographic variations in the prevalence of pathogens such as *Plesiomonas shigelloides* and *Vibrio* underscore the influence of climate change on infectious disease transmission. Co-detection of two or more organisms occurred in 14.7% of adult samples. Pathogen detections peaked in the warmer, drier mid-year months. This study highlights the significant contribution of non-notifiable pathogens to acute diarrhea burden, contextualizing local data within global epidemiological trends. Our findings carry important implications for clinical decision-making, public health policy, One Health disease prioritization, and optimization of surveillance strategies.

**IMPORTANCE:**

Acute infectious diarrhea remains a major global health issue, yet in many cases, specific etiologies are often undiagnosed or underreported. This study uses multiplex PCR panel testing to identify a broad spectrum of diarrheal pathogens from thousands of patients across public hospitals, offering one of the most comprehensive assessments in the Southeast Asian region to date. It highlights the potentially overlooked role of non-notifiable pathogens and reveals distinct epidemiological patterns in adult and pediatric populations. Furthermore, it uncovers seasonal trends and potential links between climate and disease transmission. These findings fill critical knowledge gaps, improving clinical decision-making and strengthening public health responses. Our study supports the integration of environmental and epidemiological data to optimize health planning and offers useful insights for enhancing national surveillance strategies under a One Health approach.

## INTRODUCTION

Acute gastroenteritis (AGE) constitutes a significant public health concern, with an estimated two billion cases of diarrheal disease occurring worldwide every year (1, 2). It represents a leading cause of preventable morbidity and mortality, particularly among children (3–5). Furthermore, studies have highlighted the substantial societal and economic burden associated with acute diarrhea across several Asian countries (6–8). This encompasses both direct costs, such as hospitalization and outpatient care, as well as indirect costs, including productivity losses, caregiver absenteeism, and disruption to children’s education. Overall, AGE imposes a considerable strain on health systems and broader socioeconomic development, underscoring its importance as a public health priority.

Despite Singapore’s advanced healthcare system, information on etiological agents causing AGE remains lacking. Major acute gastrointestinal (GI) pathogens worldwide include *Salmonella*, *Shigella*, *Campylobacter*, pathogenic *E. coli*, *Vibrio*, Rotavirus, and Norovirus (2). However, only certain diarrheal diseases, such as salmonellosis, campylobacteriosis, Hepatitis A, Hepatitis E, cholera, typhoid, and paratyphoid fever, are notifiable under Singapore’s Infectious Diseases Act (9). Approximately 2,000 such cases are reported annually (10). This represents only a small fraction of the acute diarrhea cases managed within Singapore’s healthcare system, which are estimated at 100,000–150,000 annually in public outpatient primary care clinics (polyclinics) alone (10). This figure underestimates the true burden, as it excludes cases managed in private primary care and acute hospitals and does not account for care-seeking behavior typical of GI illness, where only more severe cases present to healthcare settings, resulting in many mild or moderate cases going unreported. The vast majority of these cases remain without pathogen characterization because etiological identification for uncomplicated AGE is seldom done, and genomic sequencing is even rarer.

Various diagnostic methods exist for pathogen identification, including stool cultures, antigen detection assays, microscopic examination with or without staining and immunofluorescence, and polymerase chain reaction (PCR) tests (11, 12). Of these, GI multiplex PCR panels are useful molecular diagnostic tools that enable rapid and accurate testing of multiple pathogens within a single assay (13, 14), via amplification of genetic material from a patient sample. These pathogens generally include bacteria, viruses, and parasites. Specifically, the BioFire FilmArray panel, used in this study, demonstrated superior diagnostic yields, faster turnaround times, and reduced healthcare costs compared to traditional laboratory methods (15–17).

GI multiplex PCR panels have been used in Singapore for at least the past decade. Clinically, such panels may be ordered when patients present with severe or persistent diarrhea, particularly in cases when rapid diagnosis is needed to guide clinical management. For instance, parasitic or *Clostridioides difficile*-associated diarrhea requires specific therapeutics (18, 19), while antibiotics are contraindicated in Shiga-like toxin-producing *Escherichia coli* (STEC) infection due to the risk of the patient developing hemolytic-uremic syndrome (20, 21). Additionally, microbiological investigations play a vital public health role, serving as a critical tool in supporting timely and effective outbreak response. Identification of causative pathogens enables a deeper understanding of an outbreak’s origin and transmission dynamics, guiding targeted public health interventions and informing the development of control measures to mitigate further spread (22, 23).

Gaining a deeper understanding of local GI epidemiology will enhance clinical decision-making through the provision of empirical data that can inform diagnostic protocols and patient management (24). Furthermore, it supports public health surveillance and response toward AGE incidents by establishing an endemic baseline, against which atypical signals can be detected and interpreted (25). Ultimately, epidemiological findings can provide valuable insights in domains such as vaccine-preventable foodborne illnesses, antimicrobial resistance, and strategic resource allocation, including the design of targeted health education campaigns and formulation of food safety regulations (26, 27).

This study aimed to establish a baseline understanding of the local epidemiology of diarrheagenic pathogens through analysis of GI multiplex PCR data collated from public hospitals in Singapore. Specifically, its objectives were to assess sample positivity and relative abundance of various microbial pathogens, broadly describe temporal trends in hospital testing practices, and compare local findings with studies from other countries to explore potential implications on clinical and public health management.

## MATERIALS AND METHODS

### Study design

This was a retrospective multicenter study utilizing laboratory records of GI multiplex PCR panels from five public hospitals. All testing was performed as part of routine clinical care, and all data used in this study were de-identified.

### Case definition

All GI multiplex PCR panel tests performed on stool samples from patients of the participating hospitals were suitable for inclusion. To maximize data retrieval, data were gathered starting with the earliest available records wherever possible. As data were de-identified prior to collation, de-duplication by patient identifier was not possible.

Only complete 22-target BioFire FilmArray GI multiplex PCR panels, conducted on stool samples and excluding other specimen types such as rectal swabs, were included in the analysis. PCR tests to detect single pathogens, or subsets of pathogens, were excluded as these are typically follow-up or targeted tests and could bias pathogen frequency estimates.

### Data sources and data fields collected

Two datasets were collected from the microbiology laboratory services of participating public acute hospitals. The first data set comprised data from four hospitals, while the second contained data from a single hospital.

The firstdata set was compiled from the Singapore General Hospital, a large tertiary care institution, and also included specimens submitted for testing by three other participating hospitals, namely Tan Tock Seng Hospital, National University Hospital, and Changi General Hospital. These hospitals are located in different geographical regions of Singapore and collectively provide a substantial proportion of acute medical care for the general population. This data set comprised individual sample-level data from December 2016 to October 2024, largely from individuals aged 16 years and above. For each sample, the patient’s age, gender, sample collection date, and multiplex PCR result were recorded.

The second data set was compiled from KK Women’s and Children’s Hospital, a tertiary hospital with an established pediatric service. Samples in this data set were predominantly from individuals aged 0–16 years, reflecting the hospital’s general age threshold for pediatric care. This data set comprised aggregate data by week, from August 2022 to September 2024. For each week, the number of samples tested, the number of samples positive for at least one pathogen, and aggregate multiplex PCR results (counts per pathogen per week) were recorded.

Due to practical limitations, no further clinical data were obtained from the patients contributing samples in either data set.

### Laboratory processing

Specimens, including both formed and loose stool, were collected with the assistance of healthcare professionals. Following the collection, samples were transported to the laboratory immediately. Testing was performed either immediately upon receipt or after short-term storage at −80°C to preserve nucleic acid integrity. All molecular testing was conducted strictly according to the manufacturer’s instructions. Internal controls and run controls were included in each assay run to monitor performance and validity of results. Participating laboratories were accredited to ISO 15189 standards.

For both data sets, PCR testing was performed using the BioFire FilmArray GI multiplex PCR panel (BioFire Diagnostics, Salt Lake City, UT, USA). This assay detects 22 common GI pathogens, including 13 bacteria, 5 viruses, and 4 parasites. The full list of pathogens is shown in Table S1.

### Data analysis

As the patient profile differed substantially, the two datasets were analyzed separately. The first and second data sets will henceforth be referred to as the “adult data set” and “pediatric data set,” respectively.

Descriptive analysis was conducted to determine sample positivity rates and relative abundances of various pathogens. Comparison of proportions between the adult and pediatric data sets was performed using the chi-square test. The overall co-detection rate and proportion of co-detection for each pathogen were calculated for the adult data set. To explore potential associations between co-detected pathogens, pairwise correlations were assessed using the phi coefficient for each pathogen combination among samples with at least two organisms detected.

Additionally, an exploratory analysis was undertaken to investigate potential seasonal patterns and the influence of climate on pathogen detections. This involved summing the number of pathogen detections by month across the study period, both overall and for selected key pathogens. The resulting data were further overlaid with publicly available national temperature and rainfall data (28, 29), averaged monthly over the study years. Spearman’s rank correlation coefficient was calculated between the monthly number of pathogen detections and environmental variables. This analysis focused on the adult data set, given its larger sample size and longer timeframe. Finally, trends in the number of multiplex PCR tests performed over the study period were also examined.

All reported *P*-values are two-tailed, with 95% confidence intervals (CIs) calculated where appropriate. Statistical analysis was conducted using Stata v15 (StataCorp LLC).

## RESULTS

The adult data set comprised 7,543 GI multiplex PCR panels performed between December 2016 and October 2024. Of these, 3,802 (50.4%) were obtained from male patients. Mean age was 59.8 years (± 17.3 years), and 7,498 (99.4%) of patients were aged over 16 years. Only 14 samples (0.2%) yielded inconclusive results, most likely attributable to suboptimal sample quality.

The pediatric data set comprised 3,015 GI multiplex PCR panels performed between August 2022 and September 2024. While individual-level age data were unavailable, these samples were sourced from the pediatric service, which generally serves patients aged 0–16 years.

### Sample positivity

In the adult data set, 3,212 out of 7,543 samples (42.6%, 95% CI: 41.5–43.7) were positive for at least one of the 22 pathogens screened for. In the pediatric data set, this figure was 1,572 out of 3,015 samples (52.1%, 95% CI: 50.3–53.9). This yielded a statistically significant difference (*P <* 0.01) in sample positivity proportions between the two datasets.

**Fig 1:**
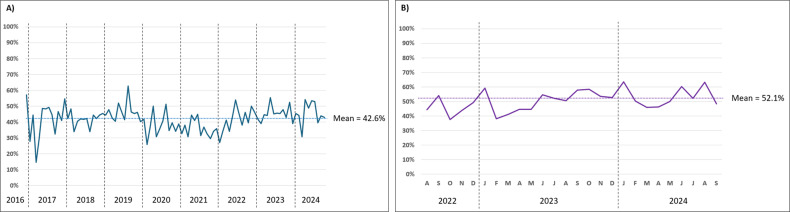
Monthly proportion of samples positive for at least one pathogen. (**A**) adult data set and (**B**) pediatric data set.

In both data sets, the monthly proportion of samples testing positive for at least one pathogen remained fairly stable throughout the respective time periods (mean percentage 42.6% ± 7.7% in the adult data set, 52.1% ± 7.0% in the pediatric data set). A visual representation of this is shown in Fig. 1A and B.

### Pathogen relative abundance

Table 1 shows the percentage of samples positive for each pathogen in the adult data set. The most frequently detected organism was enteropathogenic *E. coli* (EPEC) (17.5%, 95% CI: 16.6–18.4), followed by *C. difficile* (10.6%, 95% CI: 9.9–11.3), enteroaggregative *E. coli* (EAEC) (8.0%, 95% CI: 7.4–8.7), *Salmonella* spp. (4.3%, 95% CI: 3.8–4.8), and norovirus (4.1%, 95% CI: 3.6–4.5).

**TABLE 1:** Positivity rate (%) of pathogens detected in stool samples via GI multiplex PCR, adult data set (*n* = 7,543)

Pathogen	No. of samples positive	% of total samples^*a*^ (95% CI)
Enteropathogenic *E. coli* (EPEC)	1,320	17.5 (16.6–18.4)
*C. difficile*	797	10.6 (9.9–11.3)
Enteroaggregative *E. coli* (EAEC)	605	8.0 (7.4–8.7)
*Salmonella* spp.	323	4.3 (3.8–4.8)
Norovirus GI/GII^*b*^	307	4.1 (3.6–4.5)
*Plesiomonas shigelloides*	263	3.5 (3.1–3.9)
*Campylobacter* spp.	258	3.4 (3.0–3.9)
Enterotoxigenic *E. coli* (ETEC)	244	3.2 (2.8–3.7)
*Vibrio* spp.	176	2.3 (2.0–2.7)
Rotavirus A^*b*^	103	1.4 (1.1–1.7)
Sapovirus (I, II, IV, and V)^*b*^	62	0.8 (0.6–1.1)
Shiga-like toxin-producing *E. coli*	55	0.7 (0.5–0.9)
*Giardia lamblia* ^*c*^	48	0.6 (0.5–0.8)
*Shigella*/Enteroinvasive *E. coli*	45	0.6 (0.4–0.8)
Astrovirus^*b*^	34	0.5 (0.3–0.6)
*Vibrio cholerae*	32	0.4 (0.3–0.6)
Adenovirus F40/41^*b*^	28	0.4 (0.2–0.5)
*E. coli* O157	22	0.3 (0.2–0.4)
*Cryptosporidium* spp.^*c*^	18	0.2 (0.1–0.4)
*Cyclospora cayetanensis* ^*c*^	17	0.2 (0.1–0.4)
*Entamoeba histolytica* ^*c*^	14	0.2 (0.1–0.3)
*Yersinia enterocolitica*	5	0.1 (0.0–0.2)

^*a*^ Percentages do not add up to 100%.

^*b*^ Denotes viruses.

^*c*^ Denotes parasites.

Table 2 shows the percentage of samples positive for each pathogen in the pediatric data set. Norovirus (12.5%, 95% CI: 11.4–13.8) was the most frequently occurring organism, followed by EPEC (12.5%, 95% CI: 11.3–13.7), *Salmonella* spp. (11.0%, 95% CI: 9.9–12.1), *C. difficile* (8.9%, 95% CI: 7.9–10.0), and EAEC (7.8%, 95% CI: 6.8–8.8).

**TABLE 2:** Positivity rate (%) of pathogens detected in stool samples via GI multiplex PCR, pediatric data set (*n* = 3,015)

Pathogen	No. of samples positive	% of total samples^*a*^ (95% CI)
Norovirus GI/GII^*b*^	378	12.5 (11.4–13.8)
Enteropathogenic *E. coli* (EPEC)	376	12.5 (11.3–13.7)
*Salmonella* spp.	331	11.0 (9.9–12.1)
*C. difficile*	268	8.9 (7.9–10.0)
Enteroaggregative *E. coli* (EAEC)	234	7.8 (6.8–8.8)
Rotavirus A^*b*^	172	5.7 (4.9–6.6)
*Campylobacter* spp.	150	5.0 (4.2–5.8)
Sapovirus (I, II, IV, and V)^*b*^	113	3.7 (3.1–4.5)
Adenovirus F40/41^*b*^	91	3.0 (2.4–3.7)
Astrovirus^*b*^	70	2.3 (1.8–2.9)
Enterotoxigenic *E. coli*	52	1.7 (1.3–2.3)
*Plesiomonas shigelloides*	39	1.3 (0.9–1.8)
*Shigella*/Enteroinvasive *E. coli*	25	0.8 (0.5–1.2)
*Vibrio* spp.	20	0.7 (0.4–1.0)
Shiga-like toxin-producing *E. coli*	11	0.4 (0.2–0.7)
*Giardia lamblia* ^*c*^	10	0.3 (0.2–0.6)
*Cryptosporidium* spp.^*c*^	5	0.2 (0.1–0.4)
*E. coli* O157	2	0.1 (0.0–0.2)
*Entamoeba histolytica* ^*c*^	2	0.1 (0.0–0.2)
*Yersinia enterocolitica*	2	0.1 (0.0–0.2)
*Cyclospora cayetanensis* ^*c*^	1	0.0 (0.0–0.2)
*Vibrio cholerae*	0	0.0 (0.0–0.1)

^*a*^ Percentages do not add up to 100%.

^*b*^ Denotes viruses.

^*c*^ Denotes parasites.

Although the top five most frequently detected pathogens were the same in both data sets, their relative prevalence differed. Chi-square comparisons showed statistically significant differences in the proportions of all these pathogens except EAEC, highlighting distinct epidemiological profiles in adults and children (Table 3).

**TABLE 3:** Comparing prevalence of the most frequently occurring pathogens, adult vs pediatric data sets

Pathogen	% of total samples in adult data set (*n* = 7,543)	% of total samples in pediatric data set (*n* = 3,015)	*P*-value
Enteropathogenic *E. coli*	17.5	12.5	<0.01
*C. difficile*	10.6	8.9	<0.01
Enteroaggregative *E. coli*	8.0	7.8	0.66
*Salmonella* spp.	4.3	11.0	<0.01
Norovirus GI/GII	4.1	12.5	<0.01

To explore the impact of climate and geography on diarrheal illnesses, data from the adult data set were further compared with a large-scale US study, which likewise aggregated results from BioFire FilmArray GI multiplex PCR panels across multiple clinical laboratories (30). The comparison is presented in Table S2. While results should be interpreted with caution due to potentially different testing practices and healthcare settings, the use of a common diagnostic platform allows for informative cross-country comparisons. The most commonly detected pathogens were largely similar, although their percentage prevalences varied significantly. Notable findings include a significantly higher prevalence of *Campylobacter* in the United States compared to Singapore (5.2% vs 3.4%, *P <* 0.01), while the prevalence of *Salmonella* was similar between the two settings (4.1% vs 4.3%, *P =* 0.58). Additionally, *Plesiomonas shigelloides* was significantly more prevalent in Singapore than in the United States (3.5% vs 0.5%, *P <* 0.01), as was *Vibrio* (2.3% vs 0.3%, *P <* 0.01).

### Co-detection

In the adult data set, 1,108 out of 7,543 samples (14.7%, 95% CI: 13.9–15.5) showed co-detection of two or more pathogens from the full panel (Table 4). As individual-level data were unavailable, a corresponding figure could not be calculated for the pediatric data set.

**TABLE 4:** Total number of positive samples by number of organisms detected, adult data set

Number of organisms detected in the multiplex panel result	No. of samples	% of total (% positives)
At least one	3,212	42.6 (100)
One	2,104	27.9 (65.5)
Two	790	10.5 (24.6)
Three	229	3.0 (7.1)
Four	64	0.8 (2.0)
Five	16	0.2 (0.5)
Six	5	0.1 (0.2)
Seven	0	0.0 (0.0)
Eight	1	0.0 (0.0)
Nine	0	0.0 (0.0)
Ten	2	0.0 (0.1)
Eleven	1	0.0 (0.0)

All organisms within the panel were co-detected to some degree. For a majority of pathogens (15/22), co-detections accounted for more than 50% of their total detections. Table 5 summarizes these findings.

**TABLE 5:** Co-detected pathogens via GI multiplex PCR, adult data set (*n* = 7,543)

Pathogen	No. of co-detections	% of total samples^*a*^	% of total detections (per pathogen)
Enteropathogenic *E. coli* (EPEC)	708	9.4	53.6
Enteroaggregative *E. coli* (EAEC)	418	5.5	69.1
*C. difficile*	325	4.3	40.8
Enterotoxigenic *E. coli* (ETEC)	182	2.4	74.6
Sapovirus (I, II, IV, and V)^*b*^	171	2.3	52.9
Norovirus GI/GII^*b*^	170	2.3	55.4
*Plesiomonas shigelloides*	167	2.2	63.5
*Campylobacter* spp.	136	1.8	52.7
*Vibrio* spp.	105	1.4	59.7
Rotavirus A^*b*^	62	0.8	60.2
*Salmonella* spp.	38	0.5	69.1
Shiga-like toxin-producing *E. coli*	33	0.4	73.3
*Vibrio cholerae*	32	0.4	100.0
*Shigella*/Enteroinvasive *E. coli*	26	0.3	41.9
*Giardia lamblia* ^*c*^	24	0.3	50.0
*E. coli* O157	22	0.3	100.0
Astrovirus^*b*^	15	0.2	44.1
Adenovirus F40/41^*b*^	12	0.2	42.9
*Cryptosporidium* spp.^*c*^	11	0.1	61.1
*Entamoeba histolytica* ^*c*^	6	0.1	42.9
*Cyclospora cayetanensis* ^*c*^	5	0.1	29.4
*Yersinia enterocolitica*	4	0.1	80.0

^*a*^ Percentages do not add up to 100%.

^*b*^ Denotes viruses.

^*c*^ Denotes parasites.

**Fig 2:**
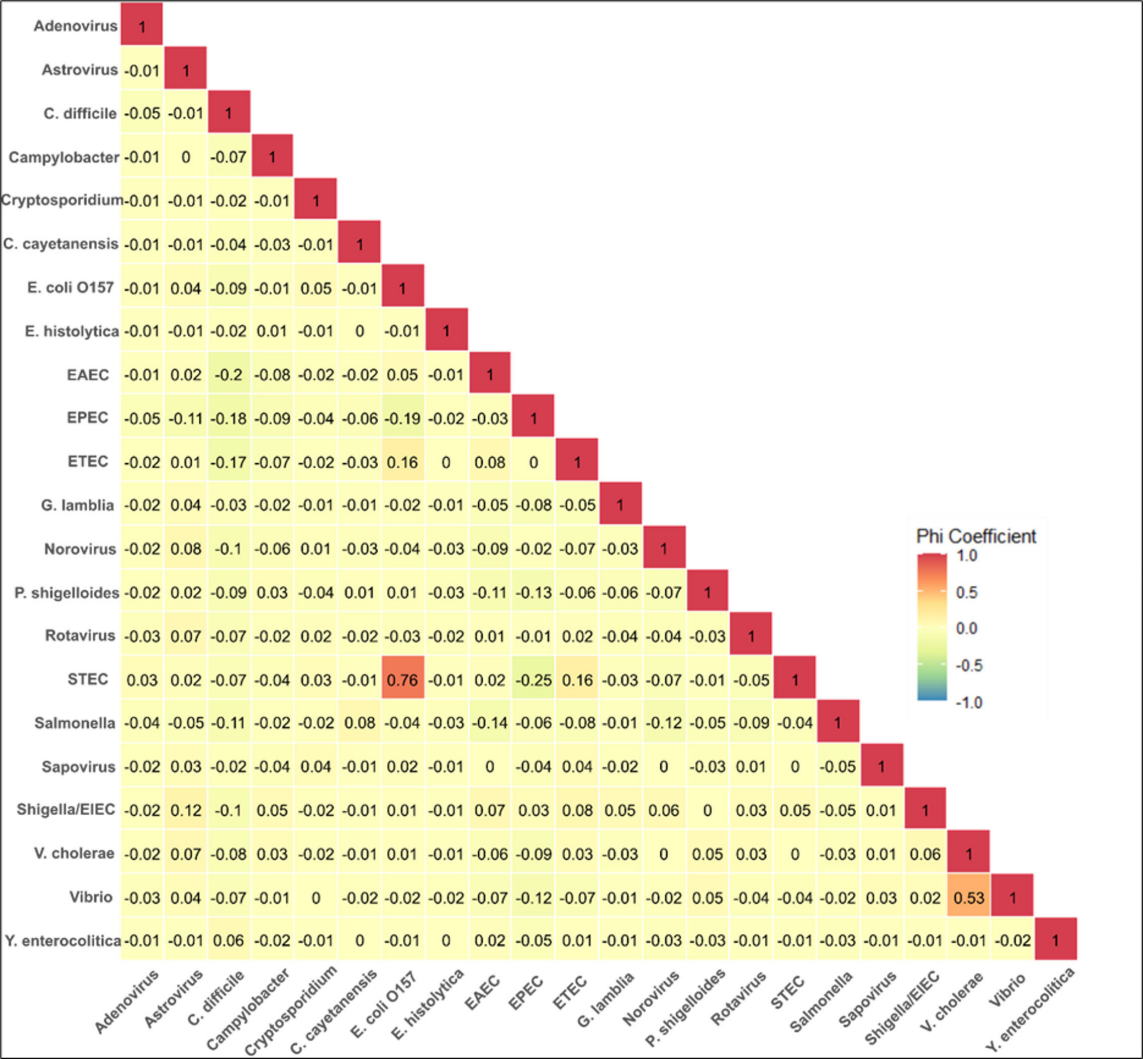
Heatmap of pathogen correlations, using samples with co-detection of two or more organisms (*n* = 1,108), adult data set. Values represent phi coefficients for each pairwise correlation.

Pairwise correlations between co-detected pathogens are shown in Fig. 2. No strong correlations were observed between any pathogen pairs (phi coefficients within ±0.3) except *E. coli* O157 and STEC (ϕ = 0.76), and *V. cholerae* and *Vibrio* spp (ϕ = 0.53). Both of these represent expected overlaps, as *E. coli* O157 is a subtype of STEC, and *V. cholerae* falls within the broader *Vibrio* genus. Weak negative correlations were noted between EPEC and STEC (ϕ = −0.25) and *C. difficile* and EAEC (ϕ = −0.20).

### Seasonal trends in pathogen detections

**Fig 3:**
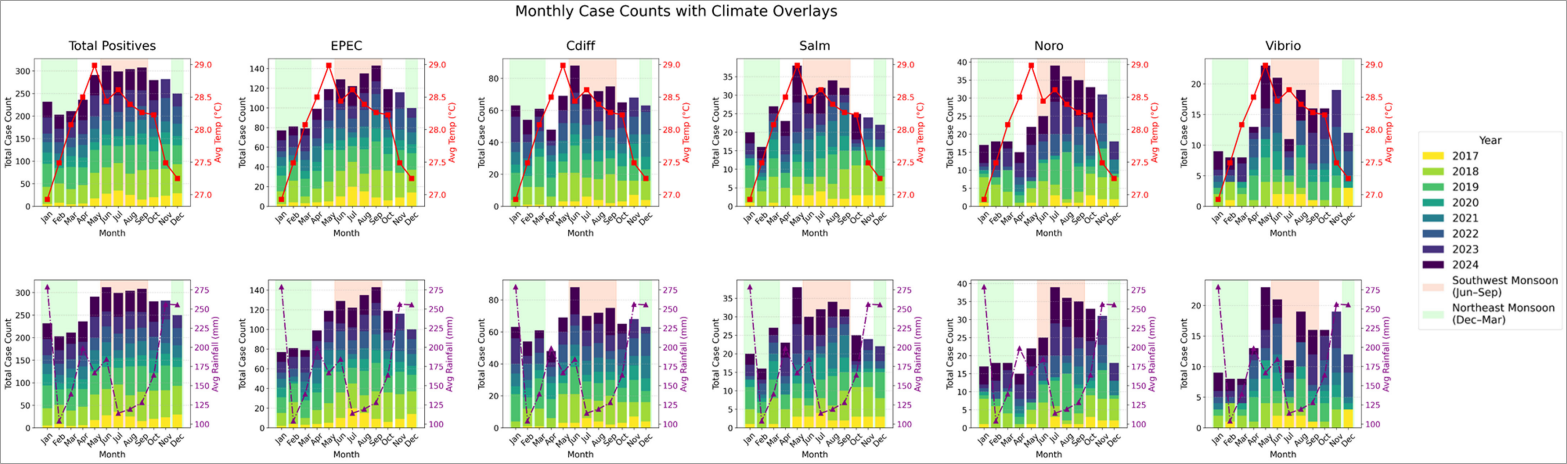
Total monthly number of pathogen detections with climate data overlays, adult data set. From left to right, the charts show counts for total positive detections, EPEC, *C. difficile*, *Salmonella*, Norovirus, and *Vibrio* spp. Average temperature data are overlaid on the top row; average rainfall data are overlaid on the bottom row.

Figure 3 presents stacked bar charts displaying the total monthly pathogen detections across various pathogens, based on the adult data set. Overall, the findings suggest a seasonal pattern in pathogen detection rates. Across the common pathogens studied, higher case counts were observed during the warmer, drier mid-year months, largely aligning with the Southwest Monsoon season (June to September). In contrast, case counts were lower during the colder, wetter year-end period corresponding to the Northeast Monsoon season (December to March). Consistent with these observations, Spearman’s correlation analysis showed a moderately strong positive correlation between total detections and temperature (ρ = 0.58), while a weak negative correlation between total detections and rainfall was observed (ρ = −0.12).

### Trends in GI multiplex PCR testing volumes

**Fig 4:**
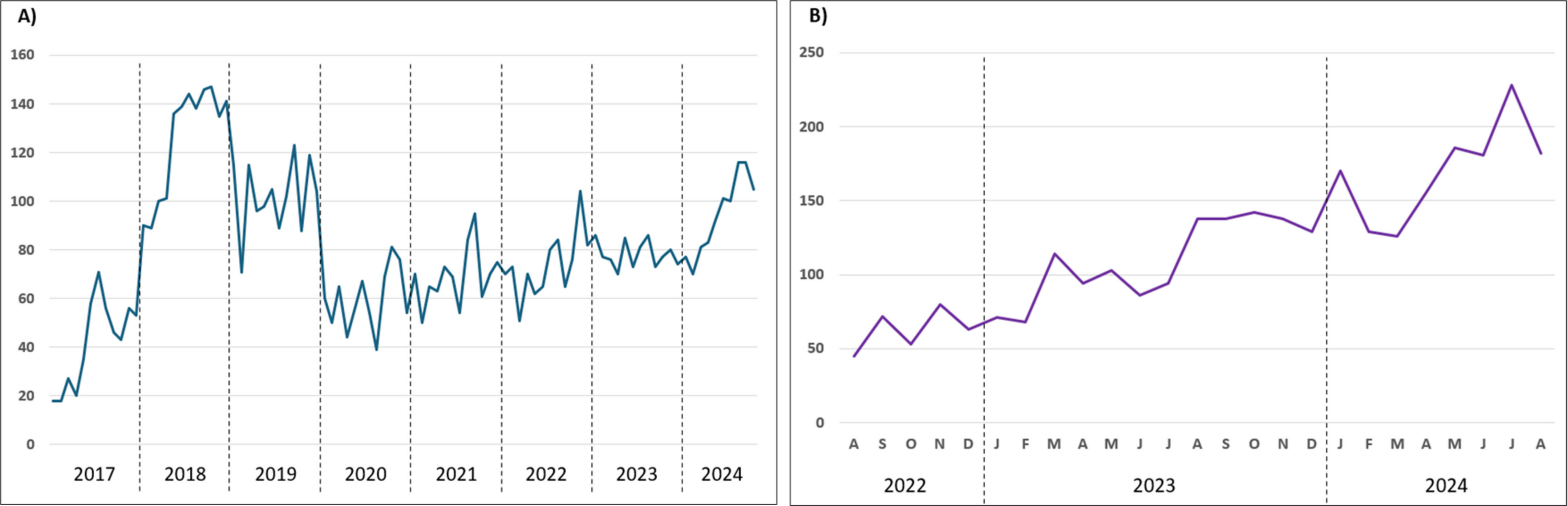
Number of multiplex panels performed each month. (**A**) adult data set and (**B**) pediatric data set. Note: Months with partial data have been excluded.

Monthly trends in the number of GI multiplex PCR panels performed are shown in Fig. 4A and B. In the adult data set, testing volumes peaked at approximately 140 per month in 2018, before declining to 40–60 per month in 2020. This was followed by a steady increase, reaching 80–100 per month by mid-2024. In contrast, the pediatric data set, covering a shorter timeframe, demonstrated a consistent upward trend, with test numbers rising from about 50 per month in August 2022 to 200 per month in mid-2024. Notably, when comparing equivalent time periods, the single pediatric hospital generally conducted more tests than the other four hospitals combined, potentially reflecting more extensive testing practices within the pediatric population.

## DISCUSSION

This study presents key findings on the epidemiology of diarrheagenic pathogens, leveraging large data sets of GI multiplex PCR panels performed over multiple years in both adult and pediatric populations.

### Sample positivity

The overall sample positivity rate of 42.6% in our adult data set exceeds the PCR-based positivity reported in a major US study (29.2%) (14), aligns closely with rates from a recent meta-analysis (39.7%) (31) and a Dutch study (39.6%) (15), but remains lower than those observed in earlier studies from Japan (52%) (32) and Germany (82%) (33). In our pediatric cohort, the sample positivity of 52.1% is significantly lower than the positivity rates reported in two studies of hospitalized pediatric diarrhea cases from France and Bulgaria (34, 35), yet higher than the overall positivity observed in a recent study from India (36). This heterogeneity in positivity rates should be interpreted with caution, as it may arise from multiple factors, including varied diagnostic techniques, case definitions, healthcare settings, and population demographics. Furthermore, the inclusion of non-infectious diarrhea cases cannot be ruled out, especially in the absence of clinical history.

### Pathogen distribution and prevalence

Regarding specific pathogen prevalence, existing literature highlights distinct epidemiological patterns of AGE in adult and pediatric populations, with viral agents being more commonly detected in children (37, 38). Our data support this finding, with the prevalence of all five viruses being higher in the pediatric data set. Nonetheless, the five most frequently detected pathogens are consistent across both data sets, underscoring the importance of prioritizing them in efforts to reduce AGE burden.

Diarrheagenic *E. coli*, particularly EPEC, was the predominant pathogen found in the adult data set. *E. coli* is one of the commonest etiologies of infective diarrhea globally (34, 39). Although *E. coli* is typically a benign commensal of the human gut microbiota, the acquisition of virulence factors through horizontal gene transfer enables specific strains to become pathogenic and cause disease (40). Pathotypes, such as EPEC and EAEC, are defined via molecular detection of these virulence genes (41). These pathogenic strains may exhibit human-to-human fecal-oral transmission and can be responsible for outbreaks (42, 43). Several studies, including a multi-country European study (44) and a 2020 systematic review (31), have also identified EPEC as the leading cause of AGE. These findings highlight the critical need to mitigate pathogenic *E. coli* transmission, particularly through comprehensive food safety protocols and hygiene measures, given its frequent presence in raw and undercooked foods (45).

*Clostridioides difficile* had a prevalence of 10.6% and 8.9% in the adult and pediatric data sets, respectively. While *C. difficile* infection (CDI) may represent asymptomatic colonization in young children (46), it is often associated with antibiotic exposure and hospitalization in adults (47). It may cause life-threatening complications such as pseudomembranous colitis, particularly in immunocompromised or elderly patients (48). New hypervirulent strains have also been responsible for outbreaks in the United States and Europe (49). A recent study reported a decline in healthcare-associated CDI in the United States; however, there is growing concern over the rising incidence of community-associated CDI, particularly among younger individuals without prior antibiotic exposure (50). While the exact reasons causing these epidemiological shifts remain unclear, there is a general consensus that robust antimicrobial stewardship remains a cornerstone of the strategy to combat this growing threat (51). A key component of such stewardship efforts is public and healthcare professional education on the responsible use of antibiotics, thereby reducing inappropriate prescribing and minimizing the negative impacts of antibiotic misuse.

*Salmonella* spp. were also frequently detected, with a prevalence of 4.3% and 11.0% in the adult and pediatric data sets, respectively. These findings are consistent with existing Asian studies identifying *Salmonella* as a pertinent cause of acute diarrhea (38, 52). A recent publication by the US Centers for Disease Control and Prevention (CDC) highlighted the substantial disease burden posed by *Campylobacter* and *Salmonella*, with annual case estimates of 1.9 million and 1.3 million, respectively (53). Table S2 suggests a significantly higher prevalence of *Campylobacter* in the United States compared to Singapore, while *Salmonella* prevalence is comparable. A higher prevalence of *Campylobacter* (11.8%) relative to *Salmonella* (8.1%) is also documented in a recent meta-analysis of primarily US and European studies (31). Our results reflect the opposite, with *Salmonella* being more prevalent than *Campylobacter*. This may be attributable to the tropical climate, with studies showing that warmer conditions can accelerate *Salmonella* proliferation in both natural environments and perishable food products (54, 55). Poultry has been highlighted as a major reservoir for *Salmonella*, contributing to human infections (56). Regardless, both these pathogens can cause severe sequelae and potentially death (57). Therefore, a proactive surveillance approach is warranted for public health reasons.

Norovirus was the predominant pathogen in the pediatric data set, with its prevalence of 12.5% aligning with previous local studies (58, 59). Although norovirus infections are typically mild and self-limiting, the virus is capable of causing large-scale outbreaks due to its exceptionally high transmissibility (60). Transmission can occur through ingestion of contaminated food or water, direct person-to-person contact, aerosolized particles, or contact with fomites (61). Following the introduction of the rotavirus vaccine, norovirus has emerged as the leading cause of pediatric AGE in many regions (62, 63), although rotavirus remains predominant in low- and middle-income countries (37). In Singapore, although the rotavirus vaccine is not part of the mandatory childhood immunization schedule, it is widely available (64). This accessibility may have contributed to reducing the prevalence of rotavirus infections, underscoring the impact of preventive measures on improving health outcomes.

Interestingly, *Plesiomonas shigelloides* and *Vibrio* spp. were relatively common, with prevalences of 3.5% and 2.3%, respectively, in the adult data set. These rates significantly exceed the approximately 1% or lower prevalence typically reported in Western countries (30, 31), although vibriosis incidence in the United States has been rising in recent years (65). Conversely, a meta-analysis of Southeast Asian data from Thailand, Indonesia, Cambodia, and Vietnam estimated the prevalence of non-cholera *Vibrio* at 5% in diarrheal patients (66). Given that both *P. shigelloides* and *Vibrio* can inhabit environmental water sources, it is not surprising that varying climate may drive regional differences in their epidemiology (67, 68), especially considering the high relevance of seafood and water exposure as risk factors in Southeast Asia. This emphasizes the considerable impact of climate change on infectious disease transmission (69) and reinforces the need for a coordinated global approach to strengthen collective preparedness. Such geographical variability also underscores the importance of conducting local, context-specific epidemiological studies.

Our adult data set exhibits a high degree of pathogen co-detection (Table 5). This is consistent with prior studies reporting that for multiple pathogens, over 50% of their total detections may occur in conjunction with other organisms (30, 70). A substantial proportion of these cases may represent genuine co-infections; however, the possibility of asymptomatic carriage cannot be excluded, particularly for organisms such as *E. coli,* which is a known gut commensal. These findings highlight the complexity of interpreting enteric disease etiology. Further research is warranted to elucidate the clinical significance of such detections. While Fig. 2 does not reveal any strong pairwise correlations of interest, it is noteworthy that *C. difficile* tends to display negative correlations with other organisms. This is plausibly due to recent antibiotic exposure, which increases susceptibility to *C. difficile* while diminishing the occurrence of other pathogens.

### Seasonal trends in pathogen detections

Singapore has a tropical climate characterized by high temperatures, high humidity, and frequent rainfall throughout the year. Nevertheless, it experiences two distinct monsoon seasons separated by inter-monsoonal periods. Higher rainfall and lower temperatures are observed in the Northeast Monsoon season from December to March, particularly in the initial wet phase. Subsequently, conditions become drier and warmer, leading into the Southwest Monsoon season from June to September (71).

 Our findings of increased pathogen detections in the warmer and drier Southwest Monsoon season (Fig. 3) are consistent with literature describing higher incidences of diarrheal illness during the hotter summer months (72–74). A prior Singaporean study similarly found that acute diarrhea attendances in primary care peaked during July and August (75). However, other studies have identified varying seasonal trends. For instance, diarrheal disease peaked during the rainy season in Bangladesh (76), winter in Shanghai (77), and autumn in Canada (78). These differences highlight that seasonal disease dynamics may be shaped by a complex network of interrelated factors, including climate, health-seeking behaviors, healthcare access, and pathogen biology. Although causality cannot be established in this preliminary analysis, the findings offer useful direction for further investigation into the mechanisms underlying these seasonal trends.

### Diagnostic testing volumes and practices

Variations in testing practices between adult and pediatric populations may also influence observed positivity rates. Our data suggest that diagnostic testing is potentially more frequently performed in children, likely due to the higher risk of severe complications such as dehydration and nutritional deficiencies if diarrhea is inadequately managed (79). However, diagnostic protocols may differ between institutions and individual clinicians as well.

Testing practices may also be influenced by external factors. Our data suggest that overall, GI multiplex panel testing in local hospitals has increased in recent years. We postulate that the reduction in sample numbers in 2020 was potentially due to the COVID-19 pandemic. Individuals may have avoided visiting acute hospitals for perceived non-urgent conditions such as diarrhea, and social distancing measures likely curtailed transmission of infectious diseases (80, 81). Furthermore, testing practices during specific time periods may have been influenced by the presence of outbreaks, where testing of asymptomatic individuals may have been pursued for active case finding. Although we are unable to determine whether such events occurred within our data sets, the stable sample positivity rate suggests they likely did not.

### Role of GI multiplex PCR panels in clinical management

Using GI multiplex PCR panels for the identification of specific pathogens offers significant benefits in clinical management. These panels enable rapid diagnosis, facilitate targeted treatment, and support the timely discontinuation of unnecessary empirical therapy (15, 82), particularly when paired with an antimicrobial stewardship program (83). Furthermore, they can enhance clinical sensitivity by detecting a wide range of pathogens from a single specimen, improving overall diagnostic efficiency (69, 84). However, recommendations on the adoption of multiplex panels should also consider their disadvantages. For instance, their overutilization in routine cases of self-limiting AGE may drive up healthcare costs without adding value to outcomes (85). Additionally, these panels do not provide information on antimicrobial susceptibility and cannot distinguish between asymptomatic carriage and active infection, potentially complicating clinical interpretation (86).

Ultimately, the decision to perform multiplex testing should be guided by clinical discretion, rather than a one-size-fits-all approach. An appreciation of the local epidemiology would help determine whether the use of these panels would contribute meaningful diagnostic insights (85). Potential benefits may also be more pronounced in specific patient populations, such as immunocompromised or clinically unstable patients (13).

### Public health implications

Our study findings may provide key evidence to guide national policy and operational decisions. Data on disease burden can inform evidence-based policymaking, such as local cost-effectiveness evaluations of rotavirus vaccination, which have been conducted in several countries (87, 88). Vaccines protective against other diarrheal pathogens, such as ETEC and cholera (89), might also be explored in the future. Regular public health messaging can also focus on the most prevalent threats. This includes raising awareness about responsible antibiotic use to mitigate rising rates of CDI. Emphasizing the importance of thorough cooking and good hygiene, particularly within the food service industry, can also help prevent outbreaks of diarrheagenic *E. coli* or *Salmonella*. Furthermore, this data can support multisectoral One Health disease prioritization efforts for zoonotic and foodborne pathogens.

Continued surveillance of diarrheagenic pathogens also remains critically important. Currently, Singapore’s national foodborne disease surveillance relies on several key data sources. These include mandatory reporting of specific notifiable diseases upon laboratory diagnosis (9), event-based surveillance via direct reporting of suspected outbreaks, and syndromic surveillance (90) via monitoring of acute diarrhea attendances at polyclinics (10). Collectively, these systems enable week-on-week tracking and prompt responses to unusual signals, thereby supporting timely public health intervention. Internationally, a similar approach combining syndromic surveillance with laboratory-based disease notifications is adopted by many countries, including the United States, United Kingdom, and South Korea (91–93).

Therefore, the data from this epidemiological study help assess whether the existing surveillance system reflects the burden of key foodborne pathogens, with implications for the scope and prioritization of notifiable infectious diseases. Broadening the range of notifiable diseases could improve situational awareness and outbreak response. However, these benefits must be weighed against the increased demands of enhanced surveillance, which could drain resources from other critical clinical or public health efforts. In fact, a recent systematic review on syndromic surveillance for GI pathogens revealed differing degrees of utility across various populations and settings, highlighting the challenges of implementing effective surveillance (94). A cost-effective recommendation could involve targeted surveillance of specific high-risk settings, such as nursing homes and childcare facilities, for early detection of potential clusters. Ultimately, surveillance systems should be context-specific and purpose-built to achieve defined objectives (95).

### Limitations

This study has a few limitations. First, clinical data—such as detailed demographics, reasons for hospital admission, and epidemiological risk factors—were not collected. Therefore, the indications for GI multiplex testing could not be determined, limiting our ability to characterize the source population. The lack of standardized testing criteria may also introduce bias in the observed relative abundance of pathogens, as clinical presentation likely influences testing decisions. For example, pathogens with milder symptoms or predominant vomiting rather than diarrhea may be underrepresented. Additionally, cases where clinicians strongly suspect a specific pathogen and opt for targeted testing instead of multiplex panels would not be captured in our data sets. The absence of de-duplication by patient identifier also limited the ability to account for repeated samples from individual patients, potentially leading to biased estimates of pathogen frequency. Conducting a dedicated study with predetermined case definitions would address this shortcoming.

Second, while the BioFire FilmArray GI multiplex PCR panel detects a broad array of 22 organisms, it is still not exhaustive for all GI pathogens. For instance, the panel excludes Hepatitis A and Hepatitis E, which are notifiable diseases under Singapore’s Infectious Diseases Act, as well as certain toxin-producing bacteria such as *Bacillus cereus* and *Staphylococcus aureus*. Thus, further studies may be needed to investigate the disease burden attributable to these pathogens.

Finally, GI multiplex PCR results should be interpreted with caution and correlated clinically where possible, as positive findings may be due to asymptomatic colonization (96, 97). False positives may also arise (98, 99). Additionally, multiplex PCR panels detect nucleic acids and do not confirm pathogen viability; therefore, positive results may reflect DNA or RNA from non-viable organisms, potentially leading to an overestimation of active infections. Nevertheless, the BioFire FilmArray GI multiplex PCR panel maintains high overall diagnostic accuracy, with reported sensitivity exceeding 94% and specificity over 97% (70). Ideally, the GI multiplex PCR results would be compared against those of a healthy control group to better assess the clinical significance of detected pathogens.

### Conclusions

In conclusion, this study presents key insights into the epidemiology of diarrheagenic pathogens across both adult and pediatric populations in Singapore’s hospitals. It highlights the significant contribution of non-notifiable pathogens to overall AGE burden, underscoring the need to address these common infectious agents. These findings carry important implications for clinical decision-making, public health policy, and optimization of surveillance strategies. Additionally, a complementary study conducted in primary care facilities will offer a deeper understanding of pathogens circulating in the community, allowing meaningful comparisons with hospital-based findings and strengthening the response to these public health threats.

## SUPPLEMENTAL MATERIAL

Supplemental material spectrum.02725-25-s0001.docx Tables S1 and S2.

ASM does not own the copyrights to Supplemental Material that may be linked to, or accessed through, an article. The authors have granted ASM a non-exclusive, world-wide license to publish the Supplemental Material files. Please contact the corresponding author directly for reuse.
