# Supplementary material for: Epidemiology of diarrheagenic pathogens in acute hospitals in Singapore: a retrospective multicenter study
Source: Microbiol Spectr. 2025 Nov 12;13(12):e02725-25. doi: 10.1128/spectrum.02725-25 (PMC12671213; doi:10.1128/spectrum.02725-25)
Supplement: Supplemental material — Tables S1 and S2. [file spectrum.02725-25-s0001.docx]

**Supplemental Materials**

For manuscript submission “Epidemiology of diarrhoeagenic pathogens in Singapore’s acute hospitals – a retrospective multicentre study”

**Table S1**. Pathogens included in the BioFire FilmArray GI multiplex PCR panel

| **Bacteria** |
| --- |
| *Campylobacter (C. jejuni, C. coli, C. upsaliensis)* |
| *Clostridioides difficile* (toxin A/B) |
| *Plesiomonas shigelloides* |
| *Salmonella* |
| *Yersinia enterocolitica* |
| *Vibrio (V. parahaemolyticus, V. vulnificus, V. cholerae)* |
| *Vibrio cholerae* |
| Enteroaggregative *E. coli* (EAEC) |
| Enteropathogenic *E. coli* (EPEC) |
| Enterotoxigenic *E. coli* (ETEC) *lt/st* |
| Shiga-like toxin-producing *E. coli* (STEC) *stx1/stx2* |
| *E. coli* O157 |
| *Shigella*/Enteroinvasive *E. coli* (EIEC) |
| **Viruses** |
| Adenovirus F40/41 |
| Astrovirus |
| Norovirus GI/GII |
| Rotavirus A |
| Sapovirus (I, II, IV, and V) |
| **Parasites** |
| *Cryptosporidium* |
| *Cyclospora cayetanensis* |
| *Entamoeba histolytica* |
| *Giardia lamblia* |

**Table S2.** Comparison between pathogen prevalences in the adult dataset (present study) and the US dataset from Ruzante et al. [1], via chi-square testing

*Viruses are highlighted in blue and parasites in green*

| Pathogen | % of total samples in adult dataset  (n = 7,543) | % of total samples in US dataset  (n = 50,192) | p-value |
| --- | --- | --- | --- |
| Enteropathogenic *E. coli* (EPEC) | 17.5 | 15.6 | <0.01 |
| *C. difficile* | 10.6 | 30.4 | <0.01 |
| Enteroaggregative *E. coli* (EAEC) | 8.0 | 6.3 | <0.01 |
| *Salmonella* spp. | 4.3 | 4.1 | 0.58 |
| Norovirus GI/GII | 4.1 | 11.1 | <0.01 |
| *Plesiomonas shigelloides* | 3.5 | 0.5 | <0.01 |
| *Campylobacter* spp. | 3.4 | 5.2 | <0.01 |
| Enterotoxigenic *E. coli* (ETEC) | 3.2 | 2.2 | <0.01 |
| *Vibrio* spp. | 2.3 | 0.3 | <0.01 |
| Rotavirus A | 1.4 | 4.1 | <0.01 |
| Sapovirus (I, II, IV, and V) | 0.8 | 4.2 | <0.01 |
| Shiga-like toxin-producing *E. coli* (STEC) | 0.7 | 1.8 | <0.01 |
| *Giardia lamblia* | 0.6 | 1.7 | <0.01 |
| *Shigella*/Enteroinvasive *E. coli* (EIEC) | 0.6 | 1.9 | <0.01 |
| Astrovirus | 0.5 | 2.7 | <0.01 |
| *Vibrio cholerae* | 0.4 | 0.5 | 0.23 |
| Adenovirus F40/41 | 0.4 | 3.0 | <0.01 |
| *E. coli* O157 | 0.3 | 0.7 | <0.01 |
| *Cryptosporidium* spp. | 0.2 | 2.2 | <0.01 |
| *Cyclospora cayetanensis* | 0.2 | 0.4 | 0.02 |
| *Entamoeba histolytica* | 0.2 | 0.1 | <0.01 |
| *Yersinia enterocolitica* | 0.1 | 1.0 | <0.01 |

References

1. Ruzante, J.M., et al., *Real-time gastrointestinal infection surveillance through a cloud-based network of clinical laboratories.* PLoS One, 2021. **16**(4): p. e0250767.
